# Typology of employers offering line manager training for mental health

**DOI:** 10.1093/occmed/kqae025

**Published:** 2024-05-09

**Authors:** H Blake, J Hassard, T Dulal-Arthur, M Wishart, S Roper, J Bourke, V Belt, C Bartle, N Pahl, S Leka, L Thomson

**Affiliations:** School of Health Sciences, University of Nottingham, Nottingham, UK; NIHR Nottingham Biomedical Research Centre, Nottingham, UK; Queen’s University Business School, Queen’s University Belfast, Belfast, Northern Ireland, UK; School of Health Sciences, University of Nottingham, Nottingham, UK; Warwick University Business School, Warwick University, Coventry, UK; Warwick University Business School, Warwick University, Coventry, UK; Cork University Business School, University College Cork, Cork, Ireland; Warwick University Business School, Warwick University, Coventry, UK; School of Medicine, University of Nottingham, Nottingham, UK; Society of Occupational Medicine, London, UK; Division of Health Research, Centre for Organisational Health & Well-being, Lancaster University, Lancaster, UK; School of Medicine, University of Nottingham, Nottingham, UK; Institute of Mental Health, Nottinghamshire Healthcare NHS Foundation Trust, Nottingham, UK

## Abstract

**Background:**

Mental ill health has a high economic impact on society and employers. National and international policy advocates line manager (LM) training in mental health as a key intervention, but little is known about employer training provisions.

**Aims:**

To explore the prevalence and characteristics of organizations that offer LM training in mental health.

**Methods:**

Secondary analysis of existing longitudinal anonymised organizational-level survey data derived from computer-assisted telephone interview surveys collected in four waves (2020:1900 firms, 2021:1551, 2022:1904, 2023:1902) in England, before, during and after a global pandemic.

**Results:**

The proportion of organizations offering LM training in mental health increased pre- to post-pandemic (2020:50%, 2023:59%) but 41% do not currently provide it. Logistic regression confirmed that LM training is more likely to be offered by large-sized enterprises, organizations with a larger proportion of employees who are younger (aged 25–49), female, disabled and from ethnic minority communities. Sector patterns were inconsistent, but in 2023, organizations from the ‘Hospitality’ and ‘Business Services’ sectors were more likely to provide LM training than other sectors.

**Conclusions:**

Continued efforts are needed to increase the proportion of employers offering LM training in mental health, particularly small- to medium-sized enterprises, and organizations with predominantly male, White and/or older workforces.

## Teaser

Line managers (LMs) play an important role in preventing workplace mental ill health but the typology of organizations providing LM training in mental health is under-researched. Secondary analysis of four-wave longitudinal firm-level survey data collected between 2020 and 2023 was undertaken to ascertain the prevalence and characteristics of organizations offering LM training. While 59% of organizations offer LM training, it is less commonly provided in small- to medium-sized enterprises and organizations with a predominantly male, White and/or older workforce.

**Key learning points**

**What is already known about this subject**

- Mental ill health has a high economic impact on society and employers.
- Line managers play an important role in preventing workplace mental ill health.
- Line manager training in mental health is recommended as a key point of intervention but the typology of organizations providing line manager training in mental health is under-researched.

**What this study adds**

- The proportion of organizations offering line manager training in mental health increased from pre- to post-pandemic (2020:50%, 2023:59%).
- However, 41% of organizations do not currently provide any LM training in mental health.
- Line manager training in mental health is less likely to be offered by small- to medium-sized enterprises and more likely to be offered by organizations with a higher proportion of employees who are younger (<49 years), female, disabled and from ethnic minority communities.

**What impact this may have on practice or policy**

- Continued efforts are needed to increase the proportion of employers offering line manager training in mental health.
- A particular focus is needed on support for the provision of line manager training in mental health in Construction, Production and Other Services industries, in small- and medium-sized enterprises, particularly micro-small organizations, and organizations with predominantly male, White and/or older workforces.

## Introduction

Incident rates of common mental health concerns (stress, anxiety and depression) are high and have been exacerbated by the coronavirus disease 2019 (COVID-19) pandemic [1,2], now accounting for over half of all work-related ill health and 17 million working days lost per year in Great Britain [1], with a high economic impact to society and employers [3,4]. The estimated total annual costs of absenteeism, presenteeism (when employees are at work but underperforming due to ill health) and labour turnover were £53–56 billion in 2020–21, which is a 25% increase since 2019, pre-pandemic [4]. The true cost of mental ill health is significantly higher when both direct and indirect costs are accounted for [3]. The non-productivity costs of poor mental health in the United Kingdom (UK), referring to intangible human costs and quality-of-life impacts, and the costs of health and service care, are estimated to be £117.9bn, approximately 5% of UK Gross Domestic Product [5].

The existence of policies and standards for mental well-being at work demonstrates a commitment to improving well-being in UK firms. The International Organization for Standardization (ISO) produced ‘ISO 45003’, which is advocated by The British Standards Institution. This was the first global standard giving practical guidance on managing psychological health in the workplace and includes guidance on the management of psychosocial risks at work which includes good people management [6]. Line managers (LMs) play a key role in protecting employee well-being as they are often the first point of contact for employees who may encounter work-related challenges or stress [7–10]. LMs are also responsible for allocating resources, managing workloads and can serve as advocates to support team members [10].

Training for LMs in mental health is now recommended by the Confederation of British Industry [11], the World Health Organization [12] and the National Institute for Health and Care Excellence [13]. However, LM training provisions are suboptimal; a survey conducted in 2019 by the Institute for Occupational Safety and Health [9] found that 57% of organizations indicated that their organization offered no mental well-being training and/or support for managerial staff. A Chartered Institute for Personnel and Development survey on ‘Health and Wellbeing at Work’ [14] found that only 25% of people professionals believe that managers within their organizations are confident and competent to spot the early warning signs of mental ill health.

Ensuring LMs are equipped with the knowledge and skills to support the prevention and management of mental ill health at work is urgent in the current context, during and beyond a period of global uncertainty and rapid changes for organizations. The aftermath of the COVID-19 pandemic led to a pronounced and prolonged deterioration in mental health [15] and catalysed a shift to remote and hybrid work patterns which may (for some) have led to increased work intensity, inability to disconnect and loneliness from reduced social interactions [16]. The current cost-of-living crisis in the UK (and beyond) further threatens immediate and longer-term mental health [17]. Although LM training in mental health is recommended as a key point of intervention [11–13], the typology of organizations providing LM training remains under-researched. An understanding of which types of organizations are not providing training could support the development and implementation of accessible and targeted training for LMs. Therefore, the aim of the study is to use an existing longitudinal data source to explore the prevalence and characteristics of organizations that offer LM training in mental health, including any differences in provision between sector, organization size or type, employee profile, and any changes over time. Insights from this study will be used to identify the typology of organizations that do, and do not, adopt this good practice for workforce well-being, and inform the targeting of LM training interventions in the future.

## Methods

Retrospective secondary analysis of anonymised firm-level survey data was conducted. Data were derived from Computer-Assisted Telephone Interview surveys collected in four waves (2020–23) as part of a broader prospective longitudinal study on workplace mental health and productivity being conducted by the same authorship team [18–21]. Telephone interviews were conducted by call centre operatives working for a market and social research organization. Interviewers were trained in research methods and completed a half-day familiarization with the surveys involving role play.

Ethical approval for this secondary analysis was granted in August 2023 by the institutional Research Ethics Committee (Ref: HSSREC-144 21-22). Data were collected from organizational representatives from non-government-funded organizations with 10 or more employees in the Midlands region of England, at each time point. The aim was to obtain as broad a response as possible during the data collection period, and so the final sample was the number of participants that responded between the survey opening and closing dates for each wave. The response rates were 17% (2020) and 15% (2021–23). Organizations participating in Wave 1 were followed up in subsequent waves by the interviewers until an appointment was made or the organization refused. However, as unbalanced panel data rather than longitudinal data were collected, new organizations were recruited at each wave to increase the overall sample size. In total, 118 organizations participated in the study across all four waves. Within each organization, the most senior person with responsibility for the health and well-being of workers was approached and invited to participate as a representative of that organization.

The first wave of survey data was collected in 2020 (1900 firms), immediately prior to the first COVID-19 lockdown in the UK, and subsequently in 2021 (1551 firms) at the height of the third national lockdown, 2022 (1904 firms) after the lifting of all social restrictions, in 2023 (1902 firms) in the final pandemic months (before pandemic end on 5 May 2023). Table 1 presents the distinct categories of survey items.

**Table 1.** Categories for survey items

Survey items	Categories	Description	Survey question
Organization size	Micro-small (1–49 employees), medium (50–249 employees), large (250+ employees)	The total number of workers currently employed	‘Do you know the approximate number of employees?’
Length of operation	0–10 years, 11–20 years, 20+ years	The number of years the organization had been operating, from its founding date to the present	For how many years has the business been operating?
Change in number of employees	Stayed the same, increased, decreased	Participants’ knowledge or perception of any apparent changes to the organization’s total number of employees in the past 12 months	Has the number of employees increased, decreased, or stayed the same over the past 12 months?
Gender distribution of employees	Female-dominated (≥75% female workforce), male-dominated (≤25% female workforce), gender-balanced (25–74% female)	Categories formed based on criteria established by Leadbeater *et al*. (2020), which, however, focused on males.	‘What proportion of staff are female?’
Age	Under 25, 25–49 years, Aged 50 and over	Proportions categorized as minimal representation (≤25%), neutral (25–74%), majority representation (≥75%)	‘What percentage of staff are aged under 25; 25–49 or 50+?’
Ethnicity	Ethnic majority (predominantly White ethnic background), ethnic minority (predominantly ethnic minority background)	The sample average for each year was used as the point of reference to categorize those organizations which fell above the sample average versus equal to or below the sample average. The sample means were 11% (2020), 12% (2021), 13% (2022) and 12% (2023).	‘What proportion of your staff are from a non-White ethnic group?’
Disability	Above sample mean, equal to or below sample mean	Sample means for disability: 2% (2020, 2021), 3% (2022), 2% (2023)	‘What percentage of your staff have a long-term disability that affects the amount or type of work they can do’
Sector	Production, construction, wholesale, retail, hospitality, business services, other services	Other services include activities of extraterritorial organizations and bodies or other services activities	
Line manager training in mental health	Yes/no		Has this activity (line manager training in mental health) taken place in the last 12 months?

To understand the typology of enterprises that offer LM training for mental health, our analytical approach was structured into three distinct parts. We first explored the characteristics of organizations offering LM training in mental health using descriptive statistics in the form of percentages and frequency counts. The focus centred on critical organizational-level variables such as sector, organization size, length of operation, change in number of employees. We also investigated characteristics of the employee profiles, namely, gender distribution, age, ethnicity and disability representation. This exploration served to provide a holistic view to elucidate what ‘type’ of organizations tend to be more involved in mental health LM training initiatives. In the subsequent phase of the analysis, we employed chi-square statistics to compare the differences in provision of mental health training between sector, organization size and employee profile. Provision of mental health training was treated as a dichotomous variable reflecting two conditions: (1) organizations providing mental health training to their LMs and (2) organizations not providing mental health training to their LMs. We conducted two binary logistic regressions to explore how the organizational characteristics and employee profiles predicted provision of mental health training. Descriptive statistics were used to assess general changes in the frequencies/percentages of these characteristics over time. This allowed us to identify trends and fluctuations in the organization attributes across the 4-year span.

## Results

**Figure 1.**
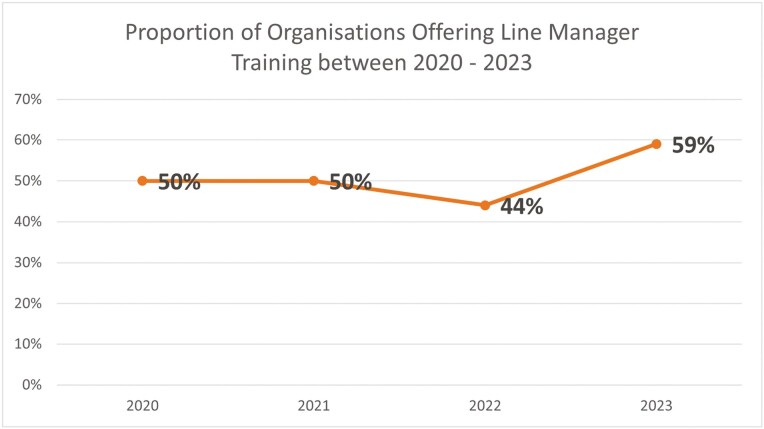
Proportion of organizations offering LM training in mental health.

The proportion of organizations offering LM training remained the same between 2020 (*n* = 413, 50%) and 2021 (*n* = 420, 50%), slightly dropped in 2022 (*n* = 371, 44%) and increased in 2023 (*n* = 470, 59%). Overall, however, the proportion of organizations offering LM training increased from pre- to post-pandemic (2020:50%, 2023: 59%), although this indicates that in 2023, 41% (*n* = 394) were still not offering their LMs any training in mental health (Figure 1).

Chi-square statistics were used to identify differences between organizations which offer LM training in mental health and those which do not, by organization size, sector and employee profile (Table 2). The total number of organizations responding at each time point was (2020, *n* = 833), (2021, *n* = 841), (2022, *n* = 952), and (2023, *n* = 970) for all variables except ‘change in number of employees’ which was (2020, *n* = 817), (2021, *n* = 838), (2022, *n* = 944), and (2023, *n* = 965). At each time point (i.e. 2020, 2021, 2022, 2023), large organizations were consistently more likely to offer LM training than small- and medium-sized enterprises (SMEs). Organizations with a larger demographic of ethnic minority employees were consistently more likely to offer LM training in mental health compared to organizations with predominantly White employees. However, exploration of effect sizes using Cramer’s V showed that the magnitude of all associations was small.

**Table 2.** Percentage of organizations which offer line manager training: variations in organization size, sector and employee profile

	2020	2021	2022	2023
Organization size	*n*(%)	*n*(%)	*n*(%)	*n*(%)
Micro-to-small	247 (45)	243 (40)	349 (49)	420 (56)
Medium	120 (56)	107 (53)	120 (59)	133 (69)
Large	46 (73)	21 (64)	28 (70)	23 (77)
*χ*^2^	*χ* ^2^(2) = 22.457***; *V* = 0.164	*χ* ^2^(2) = 15.983**; *V* = 0.138	*χ* ^2^(2) = 11.484*; *V* = 0.110	*χ* ^2^ (2) = 14.740**; *V* = 0.123
Sector	*n*(%)	*n*(%)	*n*(%)	*n*(%)
Production	53 (46)	46 (29)	65 (41)	81 (46)
Construction	35 (66)	26 (46)	28 (47)	35 (58)
Wholesale, retail	46 (45)	36 (37)	55 (49)	64 (57)
Hospitality	55 (50)	36 (54)	74 (57)	91 (71)
Business services	207 (49)	210 (48)	260 (56)	292 (63)
Other services^a^	17 (61)	17 (47)	15 (50)	13 (45)
*χ*^2^	N/S	*χ* ^2^ (5) = 24.501***, V = 0.171	*χ* ^2^ (5) = 14.129*, V = 0.122	*χ* ^2^ (5) = 26.121***, V = 0.164
Ethnic composition	*n*(%)	*n*(%)	*n*(%)	*n*(%)
Below or equal to average	218 (45)	216 (40)	290 (47)	327 (53)
Above average	195 (56)	155 (52)	207 (62)	249 (69)
*χ*^2^	*χ* ^2^(2) = 10.878**, *V* = 0.114	*χ* ^2^(2) = 11.682**, *V* = 0.118	*χ* ^2^(2) = 11.394***, *V* = 0.139	*χ* ^2^(2) = 23.525***, *V* = 0.156
Under 25	*n*(%)	*n*(%)	*n*(%)	*n*(%)
Less than 25%	214 (46)	233 (40)	312 (50)	373 (58)
25% to 49%	96 (51)	92 (48)	96 (50)	120 (66)
50%+	103 (58)	46 (47)	89 (67)	83 (60)
*χ*^2^	*χ* ^2^(2) = 7.998*, *V* = 0.098	*χ* ^2^(2) = 16.480***, *V* = 0.140	*χ* ^2^(2) = 13.414**, *V* = 0.119	N/S
Aged 25–49	*n*(%)	*n*(%)	*n*(%)	*n*(%)
Less than 25%	22 (33)	36 (51)	51 (49)	41 (49)
25% to 49%	91 (53)	89 (42)	123 (51)	146 (62)
50%+	300 (50)	246 (44)	323 (53)	389 (60)
*χ*^2^	*χ* ^2^(2) = 8.137*, *V* = 0.099	N/S	N/S	N/S
Aged 50+	*n*(%)	*n*(%)	*n*(%)	*n*(%)
Less than 25%	200 (46)	182 (47)	270 (53)	331 (64)
25% to 49%	112 (54)	105 (42)	129 (57)	152 (55)
50%+	101 (53)	84 (41)	88 (44)	93 (54)
*χ*^2^	N/S	N/S	χ2(2) = 8.7*, *V* = 0.096	χ2(2) = 8.499*, *V* = 0.094
Gender distribution	*n*(%)	*n*(%)	*n*(%)	*n*(%)
Male dominated	80 (47)	88 (37)	105 (42)	122 (46)
Gender neutral	184 (47)	147 (43)	212 (52)	256 (61)
Female dominated	149 (54)	136 (52)	180 (61)	198 (69)
*χ*^2^	N/S	*χ* ^2^(2) = 11.933**, *V* = 0.119	*χ* ^2^(2) = 19.617***, *V* = 0.144	*χ* ^2^(2) = 29.401***, *V* = 0.174
Employees with disabilities	*n*(%)	*n*(%)	*n*(%)	*n*(%)
Below or equal to average	245 (45)	241 (41)	340 (51)	385 (57)
Above average	168 (58)	130 (52)	157 (56)	191 (65)
*χ*^2^	*χ* ^2^(2) = 14.052***, *V* = 0.130	*χ* ^2^(2) = 8.151**, *V* = 0.098	N/S	*χ* ^2^(2) = 5.868*, *V* = 0.078
Change in number of employees	*n*(%)	*n*(%)	*n*(%)	*n*(%)
Increased	159 (50)	82 (41)	185 (58)	221 (61)
Decreased	36 (41)	100 (41)	82 (46)	71 (60)
Stayed the same	210 (51)	188 (48)	227 (51)	279 (58)
*χ*^2^	N/S	N/S	*χ* ^2^(2) = 6.926*, *V* = 0.086	N/S

^a^Activities of extraterritorial organizations and bodies or other services activities.

Total (*n*) refers to organizations offering LM training and those not offering training.

*P* < 0.001***, *P* < 0.01**, *P* < 0.05*.

N/S = not significant.

There were no significant differences in the proportion of organizations providing LM training in mental health by sector, length of operation, change in the number of employees, gender distribution, age distribution or disability representation, irrespective of year. The following characteristics represent the typology of organizations more likely to offer LM training: female-dominated organizations (2021, 2022, 2023), organizations with a larger portion of employees under 25 years (2020, 2021, 2022), or between 25 and 49 years (2020), organizations with a smaller proportion of employees aged 50 years or older (2022, 2023), organizations with a larger proportion of employees with disabilities (2020, 2021, 2023), and organizations where the number of employees either increased or stayed the same (2022) (Table 3). With regards to sector, there were significant differences between sectors within three of the survey waves (2021, 2022, 2023). Comparison of pre-pandemic with current data showed an increase from 2020 to 2023 in the proportion of firms providing LM training in Hospitality (+21%), Business Services (+14%), Wholesale/Retail (+12%), no change for Production, and a reduction for Other Services (−16%) and Construction (−8%).

**Table 3.** Employee profiles and provision of line manager training

						95% CI for Exp(*B*)	
Reference category		B	S.E.	Sig.	Exp(*B*)	Lower	Upper
	2020 (*n* = 833)						
Male dominated	Female dominated	0.104	0.206	0.613	0.901	0.602	1.349
	Gender balanced	0.194	0.167	0.244	0.823	0.594	1.141
Below or equal to average (disability)	Above average	0.426	0.156	0.006	0.653	0.481	0.887
Below or equal to average (ethnicity)	Above average	0.301	0.150	0.045	0.740	0.552	0.993
Under 25 Less than 25%	25–49%	0.480	0.200	0.016	0.619	0.418	0.916
	50%	0.316	0.232	0.173	0.729	0.463	1.148
25–49 Less than 25%	25–49%	0.776	0.290	0.007	0.460	0.261	0.812
	50%	−0.022	0.188	0.909	1.022	0.706	1.479
50 & over Less than 25%	25–49%	0.081	0.201	0.685	0.922	0.622	1.367
	50%	−0.221	0.226	0.327	1.247	0.802	1.941
	*χ* ^2^(8) = 38.488***; Cox and Snell *R*-Square = 0.045; Nagelkerke *R*-Square = 0.060.						
	2021 (*n* = 841)						
Male dominated	Female dominated	0.480	0.188	0.011	0.619	0.428	0.895
	Gender balanced	0.373	0.170	0.028	0.689	0.494	0.961
Below or equal to average (disability)	Above average	0.363	0.156	0.020	0.696	0.512	0.945
Below or equal to average (ethnicity)	Above average	0.432	0.151	0.004	0.649	0.482	0.874
Under 25 Less than 25%	25–49%	0.064	0.239	0.788	0.938	0.587	1.499
	50%	−0.541	0.271	0.046	1.717	1.010	2.919
25–49 Less than 25%	25–49%	−0.459	0.290	0.114	1.583	0.896	2.795
	50%	−0.017	0.195	0.932	1.017	0.694	1.489
50 and over Less than 25%	25–49%	−0.198	0.216	0.360	1.219	0.798	1.861
	50%	−0.054	0.221	0.806	1.056	0.685	1.627
	*χ* ^2^(8) = 43.394***; Cox and Snell *R*-Square = 0.050; Nagelkerke *R*-Square = 0.067.						
	2022 (*n* = 952)						
Male dominated	Female dominated	0.662	0.183	0.000	0.516	0.360	0.738
	Gender balanced	0.373	0.159	0.019	0.688	0.504	0.941
Below or equal to average (disability)	Above average	0.132	0.149	0.375	0.876	0.655	1.173
Below or equal to average (ethnicity)	Above average	0.480	0.145	0.001	0.619	0.466	0.822
Under 25 Less than 25%	25–49%	0.686	0.230	0.003	0.503	0.321	0.790
	50%	0.830	0.251	0.001	0.436	0.267	0.712
25–49 Less than 25%	25–49%	0.147	0.259	0.570	0.863	0.520	1.434
	50%	0.110	0.186	0.554	0.896	0.623	1.289
50 and over Less than 25%	25–49%	−0.327	0.217	0.154	1.355	0.893	2.056
	50%	−0.682	0.222	0.003	1.926	1.251	2.966
	*χ* ^2^(8) = 56.648***; Cox and Snell *R*-Square = 0.058; Nagelkerke *R*-Square = 0.077.						
	2023 (*n* = 970)						
Male dominated	Female dominated	0.880	0.185	0.000	0.415	0.289	0.596
	Gender balanced	0.389	0.167	0.020	0.678	0.489	0.940
Below or equal to average (disability)	Above average	0.246	0.153	0.108	0.782	0.580	1.055
Below or equal to average (ethnicity)	Above average	0.606	0.147	0.000	0.546	0.409	0.728
Under 25 Less than 25%	25–49%	−0.320	0.232	0.167	1.377	0.875	2.168
	50%	−0.457	0.256	0.074	1.580	0.956	2.609
25–49 Less than 25%	25–49%	0.322	0.274	0.240	0.725	0.424	1.239
	50%	−0.271	0.190	0.154	1.311	0.903	1.903
50 and over Less than 25%	25–49%	−0.461	0.214	0.031	1.586	1.042	2.412
	50%	−0.063	0.220	0.776	1.065	0.692	1.639
	*χ* ^2^(8) = 66.813***; Cox and Snell *R*-Square = 0.067; Nagelkerke *R*-Square = 0.090.						

Organization-level characteristics were examined using binary logistic regression. Hosmer and Lemeshow Test indicated no violations of the binary logistic assumption in all 4 years: 2020 [*χ*^2^(8) = 5.744, *P* = 0.676], 2021 [*χ*^2^(8) = 1.990, *P* = 0.960], 2022 [*χ*^2^(8) = 9.004, *P* = 0.342] and 2023 [*χ*^2^(8) = 4.113, *P* = 0.847]. Binary logistic regression was used to generate odds ratios (ORs) with 95% confidence intervals (CIs). In 2020, *organization size* was the only significant variable, with larger organizations being significantly more likely to offer LM training than micro-SMEs In 2021, 2022 and 2023, large organizations were significantly more likely to offer LM training than micro-small enterprises, but not medium-sized organizations. This indicates a slight increase in the number of medium-sized organizations offering training over the past 3 years.

In 2021, the ‘Production’ sector was significantly more likely than the ‘Other services sector’ to offer LM training, whilst in 2023, it was the ‘Hospitality and Business’ services sectors which were more likely to offer LM training (Table 4).

**Table 4.** Organizational characteristics and provision of line manager training

						95% CI for Exp(*B*)	
Reference category		B	S.E.	Sig.	Exp(*B*)	Lower	Upper
	2020 (*n* = 810)						
Stayed the same	Employees increased	−0.059	0.156	0.705	0.943	0.694	1.281
	Employees decreased	−0.373	0.244	0.126	0.688	0.427	1.110
Micro-small	Large	1.295	0.329	0.000	0.274	0.144	0.522
	Medium	0.866	0.343	0.012	0.421	0.215	0.824
20+ years	0–10 years	0.098	0.196	0.615	1.103	0.752	1.620
	11–20 years	0.055	0.169	0.745	1.056	0.758	1.472
Other	Production	−0.671	0.455	0.140	0.511	0.210	1.246
	Construction	0.075	0.510	0.884	1.077	0.396	2.928
	Wholesale	−0.615	0.463	0.184	0.541	0.218	1.339
	Hospitality	−0.369	0.458	0.420	0.691	0.282	1.697
	Business	−0.429	0.427	0.315	0.651	0.282	1.503
	*χ* ^2^(11) = 32.444**; Cox and Snell *R*-Square = 0.039; Nagelkerke *R*-Square = 0.052.						
	2021 (*n* = 835)						
Stayed the same	Employees increased	−0.217	0.182	0.234	0.805	0.564	1.151
	Employees decreased	−0.220	0.170	0.196	0.802	0.574	1.120
Micro-small	Large	1.042	0.389	0.007	0.353	0.165	0.756
	Medium	.447	0.407	0.272	0.640	0.288	1.420
20+ years	0–10 years	.056	0.198	0.777	1.058	0.717	1.560
	11–20 years	−0.218	0.175	0.212	0.804	0.571	1.132
Other	Production	−1.189	0.414	0.004	0.304	0.135	0.685
	Construction	−0.391	0.465	0.400	0.676	0.272	1.682
	Wholesale	−0.697	0.430	0.105	0.498	0.214	1.158
	Hospitality	−0.067	0.450	0.881	0.935	0.387	2.259
	Business	−0.292	0.387	0.450	0.747	0.350	1.593
	*χ* ^2^(11) = 47.722***; Cox and Snell *R*-Square = 0.056; Nagelkerke *R*-Square = 0.074.						
	2022 (*n* = 936)						
Stayed the same	Employees increased	0.223	0.153	0.144	1.250	0.927	1.686
	Employees decreased	−0.207	0.183	0.258	0.813	0.568	1.164
Micro-small	Large	1.029	0.376	0.006	0.357	0.171	0.747
	Medium	0.552	0.394	0.161	0.576	0.266	1.247
20 + years	0-10 years	0.137	0.196	0.483	1.147	0.782	1.683
	11-20 years	−0.056	0.152	0.715	0.946	0.702	1.275
Other	Production	−0.505	0.407	0.215	0.603	0.271	1.341
	Construction	−0.389	0.459	0.397	0.678	0.276	1.667
	Wholesale	−0.084	0.418	0.840	0.919	0.405	2.087
	Hospitality	0.195	0.414	0.637	1.216	0.540	2.735
	Business	.197	.384	.608	1.217	.574	2.584
	*χ* ^2^(11) = 36.784***; Cox and Snell *R*-Square = 0.039; Nagelkerke *R*-Square = 0.051.						
	2023 (*n* = 958)						
Stayed the same	Employees increased	0.099	0.148	0.501	1.104	0.827	1.475
	Employees decreased	0.056	0.214	0.792	1.058	0.696	1.608
Micro-small	Large	1.172	0.476	0.014	0.310	0.122	0.788
	Medium	0.566	0.494	0.252	0.568	0.216	1.495
20+ years	0–10 years	−0.151	0.197	0.445	0.860	0.585	1.266
	11–20 years	−0.012	0.154	0.938	0.988	0.730	1.336
Other	Production	0.017	0.408	0.966	1.017	0.457	2.264
	Construction	0.507	0.463	0.273	1.660	0.670	4.110
	Wholesale	0.568	0.424	0.180	1.765	0.768	4.056
	Hospitality	1.102	0.429	0.010	3.011	1.298	6.983
	Business	0.811	0.391	0.038	2.249	1.045	4.844
	*χ* ^2^(11) = 44.735***; Cox and Snell *R*-Square = 0.046; Nagelkerke *R*-Square = 0.062.						

Employee profile was examined using binary logistic regression. Hosmer and Lemeshow Test indicated no violations of the binary logistic assumption in 2020 [χ^2^(8) = 6.499, *P* = 0.591], 2021 [*χ*^2^(8) = 9.908, *P* = 0.272], 2022 [*χ*^2^(8) = 2.052, *P* = 0.979] and 2023 [*χ*^2^(8) = 3.189, *P* = 0.922]. Across the 4 years, organizations with a larger percentage of young employees (Under 25 and 25–49) were more likely to offer LM training. In 2021, 2022 and 2023, female-dominated organizations were more likely than both male-dominated and gender-balanced organizations to offer LM training. Organizations with mostly ethnic minority employees were more likely to offer LM training than those with mostly White employees in 2021, 2022 and 2023 (see Table 3).

## Discussion

Overall, the proportion of organizations offering LM training in mental health increased from pre-pandemic to 2023 (2020:50%, 2023:59%), but 41% of organizations do not currently provide any LM training. With regards to sector, increases from 2020 to 2023 were observed only in *Hospitality, Business Services* and *Wholesale/Retail*. The proportion of organizations providing LM training in mental health remained the same in *Production* but reduced over time, in *Other Services* and *Construction*. We found that LM training in mental health is less likely to be offered by small- to medium-sized enterprises, particularly micro-small organizations, and more likely to be offered by organizations with a higher proportion of employees who are younger (<49 years), female, disabled and from ethnic minority communities.

The increase in the proportion of organizations offering LM training post-pandemic compared to early 2020 demonstrates a clear commitment of UK firms to engage with the mental health agenda. This likely reflects a rising awareness of the economic impact of mental ill health on employers [1–5]. While the proportion of organizations not offering LM training (41% in 2023) is lower than that reported pre-pandemic by the UK Institute of Occupational Safety and Health (57% in 2019 [9]), there remains a high proportion of firms whose practices do not align with national and international recommendations for employers relating to ‘good people management’ which includes LM training in mental health [6,9,11–13]. Uptake is significantly lower in SMEs than large organizations, which is cause for concern as SMEs account for 99.9% of the total business population in the UK [22] (99% small [0–49 employees], 0.7% medium [50–249 employees], 0.1% large business [>250 employees]). While organizations of all sizes may experience challenges relating to employee well-being, in organizations with a smaller workforce and lack of dedicated Human Resource and Occupational Health Teams, pressures of managing employees with mental health concerns can be amplified [23]. This is exacerbated by SMEs often taking a ‘reactive’, rather than ‘proactive’ approach to mental health at work [24,25], despite calls to action for SME owners, industry and policy-makers to focus on primary prevention of mental health at work [7,12,13]. This issue is particularly pertinent in male-dominated industries such as *Construction*, in which the proportion of firms offering LM training in mental health reduced between 2020 and 2023, yet mental ill health in *Construction* is described as a ‘silent crisis’ [26], with 97% of construction professionals experiencing work-related stress [26], and a suicide rate 3.7% times higher than the UK national average [27].

The employee profile of those organizations offering LM training in mental health is relevant given that three survey waves were undertaken during the COVID-19 pandemic. The pandemic had a disproportionate impact on the physical and mental health of younger people, women, caregivers, people living with chronic conditions and disability (existing, or arising from long-COVID, including mental ill health) and ethnic minority communities [28–35]. It is possible that employers with a higher proportion of employees from these groups may have had greater exposure to (and therefore experience of) mental ill health in their workforce, and consequently, may have experienced greater financial impacts of mental ill health within the organization. Prior research with SMEs has shown that employers with experience of mental ill health in their organizations may have more proactive attitudes to the mental health at work agenda compared to those who have not observed mental ill health in their workforce (e.g. [25]), and could, therefore, be more likely to engage in firm-level mental health practices such as implementing LM training in mental health.

A strength of this secondary data analysis is that it allows us to provide a unique perspective on firms’ changing mental health practices from pre-pandemic to 2023, during a time of economic uncertainty and change, in a large sample of UK firms. To our knowledge, this is the first study to report the current prevalence and typology of organizations offering LM training for mental health, and therefore, findings have national and international relevance. Since organizational representatives self-selected to participate in the surveys, it is possible they are more engaged in the mental health at work agenda than non-responders, which means the proportion of firms offering LM training in mental health could be over-estimated. The study only includes firms from the Midlands region of the UK. However, the sample is large at each time point, includes firms of varying sizes from diverse industries, and firms are located in geographical areas of more or less affluence and are therefore broadly representative. A limitation is that the data available in the surveys do not provide information about the type of training offered (e.g. regarding content, delivery, duration), or who was delivering the intervention and to which groups of workers. It also does not take into account the year-to-year variability due to differences in sample size over the years. Finally, the survey items relating to ethnicity and gender are limited, since ethnic and gender majorities may vary across job roles and grades, and so more information would be required to explore such nuances.

Regarding study implications, continued efforts are needed to increase the proportion of employers offering LM training in mental health across all industries, but particularly in *Construction*, *Production* and *Other Services*, in SMEs (especially small organizations), and organizations with predominantly male, White and/or older workforces. Emerging research evidence suggests that training LM in mental health may improve their knowledge, attitudes and self-reported behaviours in supporting employees with mental health problems (e.g. [36,37], and reducing work-related sickness absence [38]). While free LM training resources in mental health and well-being are available from various professional bodies and charities in the UK (e.g. British Safety Council, MHFA England, Mind) in addition to commercially developed programmes at cost, there are few LM training programmes that have been developed using evidence-based processes, with training content relevant to the current work climate, and outcomes for employers and employees tested in a randomized trial. Our LM training programme called ‘Managing Minds at Work’ [39] meets this evidence gap and is currently being implemented within organizations as part of a randomized feasibility trial [40]. Moving forward, future research could explore whether organizations offering LM training engage in other mental health and well-being practices, and whether the provision of LM training in mental health is associated with organizational outcomes, such as sickness absence, presenteeism and business performance. This knowledge could support the business case for investment in mental health at work.

## References

[1] Health and Safety Executive. Work-Related Stress, Anxiety, or Depression Statistics in Great Britain, 2022. London: HSE, 2022.

[2] Office for National Statistics. *Coronavirus and Depression in Adults, Great Britain: July to August 2021*. https://www.ons.gov.uk/peoplepopulationandcommunity/wellbeing/articles/coronavirusanddepressioninadultsgreatbritain/julytoaugust2021

[3] Hassard J , Teoh K, Thomson L, Blake H. Understanding the cost of mental health at work: an integrative framework. In: Wall T, Cooper CL, Brough P, eds. The SAGE Handbook of Organizational Wellbeing. 1st edn. London: SAGE Publications Ltd, 2021. doi:10.4135/9781529757187.

[4] Deloitte. Mental Health and Employers: The Case for Investment, Pandemic and Beyond. Deloitte, 2022.

[5] McDaid D , Park AL, Davidson G, John A, Knifton L, McDaid S, Morton A, Thorpe L, Wilson N. The Economic Case for Investing in the Prevention Of Mental Health Conditions in the UK. London: London School of Economics and Political Science/ Mental Health Foundation, 2022.

[6] ISO Standards. *ISO 45003:2021. Occupational Health and Safety Management — Psychological Health and Safety at Work — Guidelines for Managing Psychosocial Risks*. 2021. https://www.iso.org/standard/64283.html

[7] Blake H , Hassard J, Bartle C, Thomson L. Training for Line Managers Should Focus on Primary Prevention of Mental Ill-Health at Work. Perspectives in Public Health, 2023;143:124–125. 10.1177/17579139231157528 PMC10225990 37232255

[8] Yarker J , Lewis R, Sinclair A, Michlig G, Munir F. Meta-synthesis of qualitative research on the barriers and facilitators to implementing workplace mental health interventions. SSM Mental Health 2022;2:100148.

[9] Institute for Occupational Safety and Health. White paper—workplace wellbeing: the role of line managers in promoting positive mental health. Manag Today 2019. https://iosh.com/media/zcinwdnp/workplace-wellbeing-management-today-whitepaper.pdf

[10] Montano D , Reeske A, Franke F, Hüffmeier J. Leadership, followers’ mental health and job performance in organizations: a comprehensive meta-analysis from an occupational health perspective. J Org Behav 2017;38:327–350.

[11] CBI. *Keep Mental Health Front of Mind Key Takeaways to Support Employees’ Mental Health and Wellbeing*. April 2021. https://www.cbi.org.uk/media/6461/front-of-mind-2021.pdf

[12] World Health Organization. Guidelines on Mental Health at Work. Geneva: WHO, 2022.

[13] National Institute for Health and Care Excellence. Mental Wellbeing at Work. NICE guideline [NG212]. London: National Institute for Health and Care Excellence, 2022.

[14] CIPD. *Health and Wellbeing at Work*. 26 September 2023. https://www.cipd.org/uk/knowledge/reports/health-well-being-work/

[15] Daly M , Sutin A, Robinson E. Longitudinal changes in mental health and the COVID-19 pandemic: evidence from the UK Household Longitudinal Study. Psychol Med 2022;52:2549–2558. doi:10.1017/S0033291720004432 33183370 PMC7737138

[16] Mutebi N , Hobbs A. *The Impact of Remote and Hybrid Working on Workers and Organisations* . Research Briefing, UK Parliament, 2022. https://post.parliament.uk/research-briefings/post-pb-0049/

[17] Broadbent P , Thomson R, Kopasker D et al. The public health implications of the cost-of-living crisis: outlining mechanisms and modelling consequences. Lancet Reg Health Eur. 2023;27:100585. doi:10.1016/j.lanepe.2023.100585 37035237 PMC10068020

[18] Enterprise Research Centre. *Workplace Mental Health and Covid-19: Experiences of Firms in the Midlands*. November 2020. https://www.enterpriseresearch.ac.uk/wp-content/uploads/2020/11/ERC-ResReport-Workplace-mental-health-and-Covid-19-experiences-of-firms-in-the-Midlands.pdf

[19] Enterprise Research Centre. *Workplace Mental Health in Midlands Firms 2021: Baseline Report*. September 2021. https://www.enterpriseresearch.ac.uk/wp-content/uploads/2021/09/ERC-Report-Workplace-Mental-Health-in-Midlands-Firms-2021.pdf

[20] Enterprise Research Centre. *Workplace Mental Health in Midlands Firms 2022: Baseline Report*. February 2023. https://www.enterpriseresearch.ac.uk/wp-content/uploads/2023/02/ERC-Report-WORKPLACE-MENTAL-HEALTH-IN-MIDLANDS-FIRMS-2022-BASELINE-REPORT.pdf

[21] Enterprise Research Centre. *Workplace Mental Health in Midlands Firms 2023: A Longitudinal Study*. May, 2023. https://www.enterpriseresearch.ac.uk/publications/workplace-mental-health-in-midlands-firms-2023-a-longitudinal-study/

[22] Department for Business and Trade. *Business Population Estimates for the UK and Regions 2023: Statistical Release*. Published 5 October 2023. https://www.gov.uk/government/statistics/business-population-estimates-2023/business-population-estimates-for-the-uk-and-regions-2023-statistical-release#composition-of-the-2023-business-population

[23] Irvine A , Suter J. Managing mental health problems in the workplace: are small businesses different? Empl Relat Int J. 2023;45:1161–1179. doi:10.1108/ER-09-2022-0451

[24] Benning FE , van Oostrom SH, van Nassau F, Schaap R, Anema JR, Proper KI. The implementation of preventive health measures in small- and medium-sized enterprises—a combined quantitative/qualitative study of its determinants from the perspective of enterprise representatives. Int J Environ Res Public Health 2022;19:3904. 35409587 10.3390/ijerph19073904 PMC8997761

[25] Blake H , Bullock H, Chouliara N. Enablers and barriers to mental health initiatives in construction SMEs. Occup Med (Lond) 2023;73:317–323. doi:10.1093/occmed/kqad075 37499074 PMC10540671

[26] Chartered Institute of Building (CIOB). Understanding Mental Health in the Built Environment. Bracknell: CIOB, 2020. https://www.ciob.org/industry/research/Understanding-Mental-Health-Built-Environment (8 July 2023, date last accessed).

[27] Burki T. Mental health in the construction industry. Lancet Psychiatry 2018;5:303. 29580608 10.1016/S2215-0366(18)30108-1

[28] Pierce M , Hope H, Ford T et al. Mental health before and during the COVID-19 pandemic: a longitudinal probability sample survey of the UK population. Lancet Psychiatry 2020;7:883–892. 32707037 10.1016/S2215-0366(20)30308-4 PMC7373389

[29] Bucciarelli V , Nasi M, Bianco F et al. Depression pandemic and cardiovascular risk in the COVID-19 era and long COVID syndrome: gender makes a difference. Trends Cardiovasc Med 2022;32:12–17. doi:10.1016/j.tcm.2021.09.009 34619336 PMC8490128

[30] Sachs JD , Karim SSA, Aknin L et al. The Lancet Commission on lessons for the future from the COVID-19 pandemic. Lancet Commissions 2022;400:1224–1280. doi:10.1016/S0140-6736(22)01585-9 PMC9539542 36115368

[31] Bhatia M. COVID-19 and BAME Group in the United Kingdom. Int J Commun Soc Dev. 2020;2:269–272. doi:10.1177/2516602620937878

[32] Moreno-Agostino D , Fisher HL, Goodman A et al. Long-term psychological distress trajectories and the COVID-19 pandemic in three British birth cohorts: a multi-cohort study. PLoS Med 2023;20:e1004145. doi:10.1371/journal.pmed.1004145 37014820 PMC10072377

[33] Office for National Statistics. *Coronavirus and the Social Impacts on Disabled People in Great Britain: March 2020 to December 2021*. 2021. https://www.ons.gov.uk/peoplepopulationandcommunity/healthandsocialcare/disability/articles/coronavirusandthesocialimpactsondisabledpeopleingreatbritain/march2020todecember2021

[34] Shakespeare T , Ndagire F, Seketi QE. Triple jeopardy: disabled people and the COVID-19 pandemic. Lancet (Lond) 2021;397:1331–1333. doi:10.1016/S0140-6736(21)00625-5 PMC7963443 33740474

[35] McIvor C , Vafai Y, Kelly B et al. The impact of the pandemic on mental health in ethnically diverse mothers: findings from the born in Bradford, tower hamlets and Newham COVID-19 research programmes. Int J Environ Res Public Health 2022;19:14316. doi:10.3390/ijerph192114316 36361196 PMC9655974

[36] Gayed A , Bryan BT, LaMontagne AD et al. A cluster randomized controlled trial to evaluate HeadCoach: an online mental health training programme for workplace managers. J Occup Environ Med 2019;61:545–551. 31045851 10.1097/JOM.0000000000001597

[37] Dimoff JK , Kelloway EK. With a little help from my boss: the impact of workplace mental health training on leader behaviors and employee resource utilization. J Occup Health Psychol 2019;24:4–19. 29939045 10.1037/ocp0000126

[38] Milligan-Saville JS , Tan L, Gayed A et al. Workplace mental health training for managers and its effect on sick leave in employees: a cluster randomised controlled trial. Lancet Psychiatry 2017;4:850–858. 29031935 10.1016/S2215-0366(17)30372-3

[39] Blake H , Vaughan B, Bartle C et al. Managing minds at work: development of a digital line manager training program. Int J Environ Res Public Health 2022;19:8006. doi:10.3390/ijerph19138006 35805665 PMC9266047

[40] Thomson L , Hassard J, Frost A et al. Digital training program for line managers (managing minds at work): protocol for a feasibility pilot cluster randomized controlled trial. JMIR Res Protoc 2023;12:e4875. doi:10.2196/48758 PMC10630869 37874612

